# Modern dietary pattern is prospectively associated with earlier age at menarche: data from the CHNS 1997–2015

**DOI:** 10.1186/s12937-020-00622-z

**Published:** 2020-09-09

**Authors:** Ruonan Duan, Yue Chen, Tian Qiao, Ruotong Duan, Mengxue Chen, Li Zhao, Yunhui Gong, Guo Cheng

**Affiliations:** 1 West China School of Public Health and West China Fourth Hospital, Sichuan University, Chengdu, PR China; 2 Department of Clinical Medicine, Changzhi Medical College, Changzhi, Shanxi PR China; 3 Department of Obstetrics, West China Second University Hospital, Sichuan University, Chengdu, PR China; 4 Key Laboratory of Birth Defects and Related Diseases of Women and Children (Sichuan University), Ministry of Education, Chengdu, PR China; 5 Laboratory of Molecular Translational Medicine, Center for Translational Medicine, Key Laboratory of Birth Defects and Related Diseases of Women and Children (Sichuan University), Ministry of Education, Department of Pediatrics, West China Second University Hospital, Sichuan University, Chengdu, Sichuan 610041 PR China

**Keywords:** Dietary patterns, Menarche, Cohort study, China health and nutrition survey

## Abstract

**Background:**

Early age at menarche is associated with risk of several chronic diseases. Prospective study on the association between dietary pattern and timing of menarche is sparse. We examined whether dietary patterns prior to the menarche onset were prospectively associated with menarcheal age in Chinese girls.

**Methods:**

One thousand one hundred eighteen girls aged 6–13 y in the China Health and Nutrition Survey (CHNS) with three-day 24-h recalls and information on potential confounders at baseline were included in the study. Dietary patterns were identified using principal component analysis. Age at menarche was self-reported at each survey. Cox proportional hazard regression models were performed to examine the associations of premenarcheal dietary patterns and menarcheal timing. Hazard ratios (HRs) and 95% confidence intervals (CIs) were calculated.

**Results:**

Three major dietary patterns were identified: modern dietary pattern, animal food pattern, and snack food pattern. After adjustment for age at baseline, region, ethnicity, maternal education level, energy intake at baseline, and body mass index Z-score at baseline, girls in the highest quartile of modern dietary pattern score had a 33% higher probability of experiencing menarche at an earlier age than those in the lowest quartile (HR: 1.33, 95% CI: 1.002–1.77, *p* for trend = 0.03). No significant association was found for the animal food pattern or snack food pattern.

**Conclusions:**

Higher adherence to modern dietary pattern during childhood is associated with an earlier menarcheal age. This association was independent of premenarcheal body size.

## Background

Early age at menarche is a risk factor for insulin resistance [1], type 2 diabetes [2, 3], cardiovascular diseases [4] and hormone-related cancers [5–7]. Data from previous studies have consistently pointed to the fact that there appears to be a trend towards earlier attainment of menarche among Chinese girls [8, 9]. Considering the potentially adverse consequences of early menarche for health in later life, identifying modifiable factors influencing the timing of menarche is of major public health relevance.

Large numbers of observational studies have addressed the role of dietary factors for menarche onset. Girls with higher intakes of fat [10] and animal protein [11, 12] had earlier menarche, while those with higher intakes of isoflavone [13], dietary fiber [14, 15] and vegetable protein [12] experienced their menarche at a later age. However, most of the existing studies focused on a single or a few nutrients or food groups. It is conceivable that nutrients and/or foods may influence menarche onset through their combined effects. Dietary pattern analysis, which examines the effects of overall diet, may be more predictive of disease risks than individual nutrients or foods [16]. To date, only two prospective observational studies conducted in Western children [17, 18] have investigated the longitudinal effects of dietary patterns on pubertal development. However, as general genetic background, food supplies, dietary patterns, and age at menarche vary by country and population, generalizability of those existing findings to a population of Chinese girls is uncertain.

In recent decades, China has experienced a remarkable transition in the structure of food consumption, such as increased consumption of animal-source foods, food away from home, and declining consumption of coarse grains and legumes [19]. Knowledge on specific type of dietary patterns might influence age at menarche among Chinese girls is lacking. A recent cross-sectional study of children in Shanghai found that unhealthy diet pattern, characterized by high intakes of dessert/snacks, soft drinks, and fried food, was positively associated with precocious puberty in girls [20]. However, this study was cross-sectional, and did not adjust for other potential confounding factors, such as energy intake. Using prospectively collected data from the China Health and Nutrition Survey (CHNS), we thus aimed to examine the prospective associations between dietary patterns during childhood and timing of menarche in Chinese girls.

## Methods

### Study population

We used data from the recent seven waves (1997, 2000, 2004, 2006, 2009, 2011 and 2015) of the CHNS, an ongoing longitudinal cohort study which was started in 1989. Details on the study protocol have been described elsewhere [21]. In brief, a multistage, random cluster procedure was used to obtain nationally representative information on economy, socio-demography, nutrition, lifestyle and health issues in both urban and rural areas in 15 provinces and municipal cities in China. The CHNS is a community and household based study. In each community, 20 households were randomly selected, and all household members were invited to participate in the study. The study was approved by the Institutional Review Board at the University of North Carolina and the National Institute of Nutrition and Health, Chinese Center for Disease Control and Prevention. All parents provided written informed consent for their children’s participation in the survey.

**Fig. 1:**
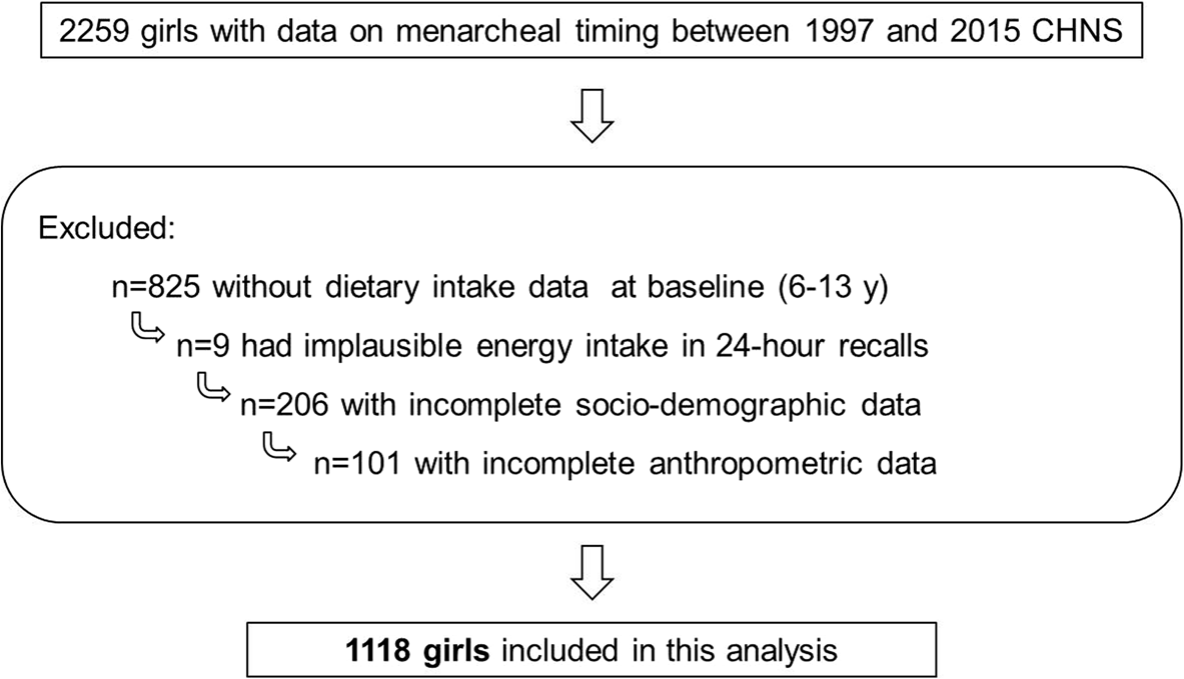
Flow chart for the study sample

Between the 1997 and the 2015 survey, there were 2259 girls with plausible data on menarche. Of these, 1434 girls aged 6–13 y who had baseline dietary information and at least one follow-up visit of menarche onset thereafter were included. We excluded 9 girls who provided dietary records with extremely low or high total energy intake values (< 400 kcal/d or > 4000 kcal/d) [13]. Three hundred seven participants were further excluded due to incomplete socio-demographic (*n* = 206) and anthropometric (*n* = 101) data. Finally, the present analysis was based on a sample of 1118 girls (Fig. 1).

### Dietary intake data

Dietary intake data of girls in the CHNS were collected by trained investigators using three consecutive 24-h recalls. When girls were 12 years or older, they were asked to recall their consumption of all foods and beverages. For girls < 12 y of age, their parents or guardians provided the information on food consumption at home, while girls provided the dietary intake information away from home. Food models and picture aids were used to improve the accuracy of the portion-size estimates [22]. The majority of the participants had a complete three-day 24-h recalls (*n* = 1098, 98.2%, i.e. 49.0% had three weekdays, 27.5% had two weekdays and one weekend day, and 21.7% had one weekday and two weekend days); the reminder had either one or two recalls (*n* = 4 and *n* = 16, respectively). Food intake was converted into energy and nutrient intake data using the Chinese Food Composition Tables (FCT) [23–26]. FCT 1991 was used in the 1997 and 2000 dietary survey; FCT 2002, FCT 2004 and 2009 (combined) were used in the 2004, 2006, 2009, and 2011 dietary survey.

### Dietary pattern derivation

Dietary intake data were divided into 18 categories (Table 1) based on their similarity in nutrient profiles and Chinese Food Composition Tables. Principal components analysis (PCA) was conducted to identify dietary patterns at baseline through the PROC FACTOR procedure in SAS software (version 9.3, SAS Institute Inc., Cary, NC, USA.). Results of the Kaiser-Meyer-Olkin test (0.65) and the Bartlett’s test of sphericity (*p* < 0.0001) indicated that the present food intake data were suitable for factor analysis. Factors were rotated orthogonally to simplify the interpretation. Based on the eigenvalues (> 1), the inspection of scree plot, and the interpretability of the factors, three factors (dietary patterns) were retained. The factor loadings represent the correlations of each food group with the corresponding dietary pattern. Food groups with factor loadings > 0.30 or < − 0.30 were considered to be strongly associated within a pattern, and thus were selected to describe the dietary patterns. Labeling of the factors was primarily descriptive, and was based on our interpretation of the pattern structures [27]. Furthermore, the factor scores of each dietary pattern were calculated for each participant by summing the product of a standardized gram of each item consumed by its factor loading [28], with a higher factor score indicating a higher adherence to the corresponding dietary pattern.

**Table 1:** Food or food groups used in the dietary pattern analysis

Food or food groups	Food items
Cereals	Rice, noodle, steamed bun, corn, barley, millet, brown rice, black rice
Tubers and starches	Potato, sweet potato, cassava, konjac powder, vermicelli
Legumes and its products	Dried legumes, tofu, soya-bean milk, dried bean curd, mung bean, red bean
Vegetables	Root vegetable, leguminous vegetable and sprout, cucurbitaceous and solanaceous vegetable, steam, leafy and flowering vegetable, aquatic vegetable
Fungi and algae	Mushroom, agaric, tremella, laver, sea-tangle
Fruits	Kernel fruit, drupe fruit, berry, orange fruit, tropic fruit, melons
Nuts	Walnut, melon seeds, cashew, hazelnut, almond, pistachio
Meat and its products	Pork, beef, mutton, rabbit meat, processed pork, sausage
Poultry and its products	Chicken, duck, goose, turkey, pigeon
Dairy products	Milk, dried milk, yoghurt, cheese
Eggs	Chicken egg, duck egg, goose egg, partridge egg
Fish and shellfish	Fish, shrimp, crab, shellfish
Ethnic foods and cakes	Pancake, tangyuan, spring rolls, mooncake, tea-oil tree, mung bean cake
Fast foods	Hamburger, sandwich, hotdog, chips, instant noodles, bread, biscuit, snacks
Beverages	Carbonated drink, fruit juice, vegetable juice, milk drink, vegetable protein drink, tea drink, powdered drink, popsicle and ice cream
Sugar and preserves	Lollipops, hard candy, chocolate, filled candy, honey, preserved fruit
Fats and oils	Animal fat, vegetable oils
Condiments	Sauce, vinegar, catsup, fermented soybean curd, pickles, spice, salt

### Assessment of age at menarche

Girls aged 8 years or older and/or their parents were asked whether menarche had already occurred during each survey, and if they had, the month and year of their first menstrual period was recorded. If girls provided different menarcheal ages in different survey years, only the first reported menarcheal age in the panel data were used for analysis to reduce potential recall bias. For the present analysis, our outcome of interest was the time for the participants to experience menarche. Thus, for girls who experienced menarche during the follow-up survey, the observation time interval was from baseline to age of first menstrual period. For girls who did not reach menarche during the follow-ups, they were censored at the last follow-up visit, i.e. the observation time was from baseline to the last follow-up visit date.

### Covariates

Detailed information on participants’ socio-demographic characteristics was collected using a structured questionnaire at baseline, including birth year, ethnicity (Han and minority), residency (urban and rural), region (northeastern area: Liaoning, Heilongjiang; east coast area: Beijing, Jiangsu, Shandong, Shanghai; central area: Henan, Hubei, Hunan; and western area: Chongqing, Guangxi, Guizhou), household income (continuous variable), and maternal education level (illiterate, primary school, middle school, high school, technical or vocational degree, and college degree or higher).

Anthropometric measurements of the participants were performed at each visit by trained research assistants according to standard procedures, with the girls dressed in underwear only and barefoot. Height and weight were measured to the nearest 0.1 cm and 0.1 kg, respectively. Body Mass Index (BMI) was calculated as weight divided by the square of height (kg/m^2^). Age-specific BMI Z-score were calculated for each participant using the equation by Cole et al. [29] based on a Chinese reference population [30].

### Statistical analysis

All statistical analyses were performed with SAS procedures (version 9.3, 2011, SAS Institute Inc., Cary, NC, USA.). Results were considered statistically significant when a two-sided *p*-value < 0.05.

We performed time-to-event analysis to investigate the prospective relevance of dietary pattern scores at baseline with the event of menarche using Cox proportional hazard regression models (PROC PHREG procedure in SAS software), which appropriately account for both the information on age at menarche from postmenarcheal girls and the censoring information from premenarcheal girls. The independent variables in the Cox proportional hazard regression models were the quartiles of each dietary pattern factor score. Hazard ratios (HRs) and 95% confidence intervals (CIs) were calculated by comparing the second, third and fourth quartiles to the first quartile (as the reference category) of each dietary pattern factor score. Also, the associations between the three dietary pattern scores on a continuous scale and menarche onset were examined. Three models were used in our study: model 1 adjusted for age at baseline, region, ethnicity and maternal education level; model 2 further adjusted for energy intake at baseline. As we were interested in the potential mediating effect of body size on diet-menarche relations, we further adjusted for BMI Z-score at baseline in model 3.

### Sensitivity analysis

Considering household income is a most frequently used proxy of socioeconomic status in investigating diet-menarche relations, we conducted sensitivity analysis with substitution of household income per capita (continuous variable) for maternal educational level in order to obtain more comparable results.

## Results

### Characteristics of study participants

General characteristics of the study sample are shown in Table 2. Girls included in the present analyses (*n* = 1118) were 8.3 ± 1.8 years old at baseline. Among them, 711 participants (63.6%) reported menarche during follow-up, and 407 participants (36.4%) were censored at the time of last follow-up visit. Overall, the participants were followed up for 4.0 ± 1.8 years after study baseline. The mean length of follow-up was longer for girls who had reached menarche (4.1 ± 1.8 y) during the follow-up than their counterparts who were censored (3.8 ± 1.7 y). The mean menarcheal age from 711 postmenarcheal girls was 12.7 ± 1.2 years. Age at menarche did not differ between the 711 girls and the 656 postmenarcheal girls who also had data on menarcheal age but were excluded from the final analysis due to lack of dietary intake at baseline, socio-demographic and anthropometric data (*p* = 0.53).

**Table 2:** General characteristics of the CHNS participants in the present study

Characteristics	Values (mean ± SD/n (%))
n	1118
postmenarcheal girls during follow-up, n	711 (63.6)
Age at baseline ^a^, y	8.3 ± 1.8
Years of follow-up, y	4.0 ± 1.8
Wave (baseline survey year)	
1997	488 (43.6)
2000	170 (15.2)
2004	177 (15.8)
2006	66 (5.9)
2009	100 (8.9)
2011	117 (10.5)
Region ^b^	
Northeastern area	199 (17.8)
East coast	210 (18.8)
Central area	359 (32.1)
Western area	350 (31.3)
Residency	
Urban	343 (30.7)
Rural	775 (69.3)
Ethnicity	
Han	950 (85.0)
Minority	168 (15.0)
Maternal education level	
Illiterate	194 (17.4)
Primary school	283 (25.3)
Middle school	404 (36.1)
High school	140 (12.5)
Technical or vocational degree	45 (4.0)
College degree or higher	52 (4.7)
Energy intake at baseline, kcal/d	1546 ± 495
Protein, % of energy	12.4 ± 3.0
Fat, % of energy	28.1 ± 11.8
Carbohydrate, % of energy	59.5 ± 12.3
Weight, kg	25.9 ± 7.3
Height, cm	127.2 ± 12.4
BMI Z-score at baseline ^c^	−0.04 ± 1.22

^a^ Mean age at recruitment

^b^ Twelve provinces were categorized into four regions: central area (Henan, Hubei, Hunan), east coast (Beijing, Jiangsu, Shandong, Shanghai), northeastern area (Liaoning, Heilongjiang), and western area (Chongqing, Guangxi, Guizhou)

^c^ BMI Z-score was calculated using the equation by Cole et al. [29] based on a Chinese reference population [30]

### Dietary patterns based on principal component analysis

The factor loadings for the three main dietary patterns are shown in Table 3. Factor 1 (*the modern dietary pattern*) was characterized by high intakes of fast foods, dairy products, fruits and eggs, and low intakes of cereals, vegetables, and condiments. Factor 2 (*the animal food pattern*) was loaded heavily for meat, poultry, fish and shellfish. Factor 3 (*the snack food pattern*) was marked by high intakes of nuts, beverages, ethnic foods and cakes, and legumes. These three dietary patterns explained 27.2% of the total variation in dietary intake (13.1, 7.5 and 6.6% for factor 1, factor 2 and factor 3, respectively).

**Table 3:** Orthogonally rotated factor loadings for three dietary patterns derived from principal components analysis ^a^

Food or food groups	Factor 1: Modern	Factor 2: Animal food	Factor 3: Snack food
Cereals	**−0.63**	0.10	−0.12
Tubers and starches	0.06	−0.21	0.00
Legumes	−0.11	−0.06	**0.40**
Vegetables	**−0.53**	0.21	−0.13
Fungi and algae	0.14	0.30	−0.04
Fruits	**0.31**	0.21	0.11
Nuts	−0.11	0.10	**0.68**
Meat and its products	0.10	**0.53**	0.05
Poultry and its products	0.21	**0.57**	−0.08
Dairy products	**0.53**	0.25	0.19
Eggs	**0.31**	0.25	0.22
Fish and shellfish	−0.02	**0.53**	0.17
Ethnic foods and cakes	0.25	−0.03	**0.45**
Fast foods	**0.63**	0.20	−0.11
Beverages	0.15	0.03	**0.48**
Sugar and preserves	0.24	0.11	−0.07
Fats and oils	0.01	−0.20	0.15
Condiments	**−0.38**	0.22	0.17
% Variance explained	13.1%	7.5%	6.6%

^a^ The bold font was used for factor loadings > 0.30 or < −0.30

### Prospective associations between three dietary patterns and menarche

Cox proportional hazard regression models for the associations between the three dietary pattern scores at baseline and age at menarche are presented in Table 4. There was a positive association between the modern dietary pattern score and probability of earlier menarche, which remained significant when the potential mediator BMI Z-score at baseline was included in the final model (adjusted HR in model 3: 1.13, 95% CI: 1.03–1.24). After adjustment for age at baseline, region, ethnicity, maternal education level, energy intake at baseline, and BMI Z-score at baseline (model 3), girls in the highest quartile of modern dietary pattern score had a 33% higher probability of experiencing menarche at an earlier age than those in the lowest quartile (adjusted HR: 1.33, 95% CI: 1.002–1.77, *p* for trend = 0.03). However, no significant association was observed for animal food pattern or snack food pattern with timing of menarche. In sensitivity analyses, replacing maternal education level with household income per capita did not change these results (data not shown).

**Table 4:** Cox proportional hazard regression models of three dietary patterns and timing of menarche among 1118 girls in the CHNS ^a^

	Model 1 ^b^	Model 2 ^c^	Model 3 ^d^
**Modern dietary pattern**			
Quartile 1	1.00	1.00	1.00
Quartile 2	0.98 (0.79, 1.21)	1.04 (0.83, 1.30)	1.03 (0.82, 1.28)
Quartile 3	1.19 (0.95, 1.50)	1.27 (0.98, 1.65)	1.24 (0.96, 1.60)
Quartile 4	1.23 (0.95, 1.59)	1.34 (1.01, 1.78)	1.33 (1.002, 1.77)
Dietary pattern score (continuous)	1.13 (1.03, 1.23)	1.14 (1.04, 1.25)	1.13 (1.03, 1.24)
*p* for trend ^e^	0.03	0.02	0.03
**Animal food pattern**			
Quartile 1	1.00	1.00	1.00
Quartile 2	1.05 (0.84, 1.30)	1.07 (0.85, 1.33)	1.06 (0.85, 1.32)
Quartile 3	1.00 (0.80, 1.25)	1.02 (0.81, 1.28)	1.01 (0.80, 1.27)
Quartile 4	1.12 (0.89, 1.41)	1.14 (0.88, 1.47)	1.12 (0.87, 1.45)
Dietary pattern score (continuous)	1.03 (0.95, 1.11)	1.03 (0.95, 1.12)	1.03 (0.95, 1.12)
*p* for trend ^e^	0.49	0.52	0.54
**Snack food pattern**			
Quartile 1	1.00	1.00	1.00
Quartile 2	0.99 (0.81, 1.23)	1.00 (0.81, 1.23)	1.00 (0.81, 1.24)
Quartile 3	0.91 (0.73, 1.13)	0.91 (0.73, 1.13)	0.91 (0.74, 1.14)
Quartile 4	0.85 (0.68, 1.06)	0.85 (0.68, 1.06)	0.85 (0.68, 1.06)
Dietary pattern score (continuous)	0.93 (0.85, 1.02)	0.93 (0.85, 1.02)	0.93 (0.85, 1.01)
*p* for trend ^e^	0.12	0.12	0.13

^a^ Values are hazard ratios (HRs) and 95% confidence intervals (95% CIs)

^b^ Model 1 adjusted for age at baseline, region, ethnicity and maternal education level

^c^ Model 2 adjusted for variables in model 1 and energy intake at baseline

^d^ Model 3 adjusted for variables in model 2 and BMI Z-score (continuous) at baseline

^e^ *P* for trends were tested with the quartiles of the three dietary pattern scores as continuous variables in the Cox proportional hazard regression models

## Discussion

In the present study, we identified three major dietary patterns in the years preceding onset of menarche among Chinese girls: modern dietary pattern, animal food pattern and snack food pattern. We found that higher adherence to modern dietary pattern was associated with higher odds of experiencing menarche at an earlier age. This association was independent of potential sociodemographic confounders and premenarcheal body size. However, no significant association was found for animal food pattern or snack food pattern.

To our knowledge, our study is the first prospective study of the associations between dietary patterns and timing of menarche in a Chinese population. Compared with studies focusing on single nutrients or foods, dietary pattern takes the interactions of nutrients or foods into account, and thus could have important public health implications because overall patterns of dietary intake might be easier for the public to translate into daily diets. It serves as a complementary approach to traditional analysis, and evidence could be enhanced when the results from multiple lines of research (i.e. nutrients, foods, and dietary patterns) are consistent [16].

The modern dietary pattern we identified showed some similarities with results previously reported by Zhang et al. [31], which was characterized by high intakes of fast foods, milk, fruits and eggs, and low intakes of grains and vegetables. We observed that modern dietary pattern was associated with an increased risk for accelerated menarche. This pattern was implicated in the timing of menarche probably in any of the three ways: high consumption of milk, high fat intake, and low consumption of plant foods. Previous studies have shown that consumption of milk contributed to high IGF-I concentrations in prepubertal children [32, 33], which was found to be associated with earlier menarche [34]. Also, high fat intake may be implicated in the earlier menarcheal timing due to its potential impact on estrogen metabolism [35]. In addition, intake of vegetable protein sources has been related to the delayed onset of puberty probably due to the high content of dietary fiber and isoflavones [35]. Taken together, these evidence suggest that modern dietary pattern has potential influence on menarcheal timing through metabolic changes in insulin-mediated pathway mechanisms and upregulation of hormones [20]. Since mid-1990s, a marked transition to the modern dietary pattern with increased consumption of sugary and fat-rich foods and declining consumption of coarse grains has occurred in China [22]. Ouyang el al [36]. reported that fruits, milk, and fast foods were the three most consumed snacks among Chinese children. Moreover, calories from fast foods account for 26–40% of total energy intake. Although more research is needed to determine the effects of modern dietary pattern on Chinese children’s health, the present public health recommendations include the necessity to limit consumption of fast foods and to increase consumption of plant foods.

It is of note that the animal food pattern was not associated with age at menarche in the present study, although most of the previous studies have shown that girls with high intake of animal protein (especially red meat) reached menarche at an earlier age [37–40]. However, some evidence has indicated that animal protein intake or meat intake was not associated with timing of menarche [41, 42]. For example, using data from the Growing Up Today Study (GUTS), Carwile et al. [41] found that peripubertal total meat or red meat intake was not related to age at menarche in 5583 US girls aged 9–14 years, which was consistent with our finding. The discrepancies across different studies suggest that the effect of animal food groups on timing of menarche may be age-specific. In fact, some researchers have proposed that animal food intake at younger ages (e.g. early childhood) may be more relevant to occurrence of menarche than peripubertal intake [43]. However, the underlying mechanisms to illustrate these potential age-specific effects remain unknown. Another potential explanation is that the animal food pattern in our study was loaded heavily for both red meat and fish/shellfish, and these two types of meat may be associated with timing of menarche in opposite ways. For example, a prospective study conducted in 456 US girls aged 5–12 y showed that red meat was inversely related to age at menarche while tuna/sardine intake was positively associated with age at menarche [40]. Similarly, we speculate that the non-significant association for snack food pattern could potentially be driven by the counteraction of positive impact of nuts and legumes which are rich in vegetable protein, dietary fiber and isoflavones [12–14, 44], and negative impact of sugary-rich foods (i.e. beverages, ethnic foods and cakes) on menarcheal timing [45, 46]. This may hence reflect a combined effect of different types of foods on menarche. However, the underlying mechanisms of the impact of snack food pattern on menarcheal timing require further study.

Our study has several strengths, including the prospective design and the representative sample from four different regions in China. Dietary intake data were collected by using a validated three-day 24-h dietary recalls. A further advantage lies in the use of dietary pattern analysis, which examines the effects of diet as a whole, and might be much easier for the public to interpret or translate into diets. In addition, the comprehensive and detailed data allowed us to simultaneously take a number of potential confounders or mediators into account and thus to reliably examine the association between dietary patterns and menarcheal timing.

Some limitations should be mentioned. First, menarche represents a relatively late stage of pubertal development. Although using menarcheal age as an indicator of puberty timing is reliable, the effects of dietary patterns on earlier events of pubertal development may differ from those on age at menarche. Future work should address the relevance of dietary patterns and early stage of puberty, such as the age at take-off (ATO, i.e. the age at minimal height velocity) [47]. Second, although the potential mediating effect of body size on diet-menarche relations was examined in our study, only BMI Z-score at baseline was considered instead of continuous change of body composition during the entire pubertal period. Furthermore, as data on parental pubertal characteristics (e.g. maternal age at menarche) is not available in the CHNS, genetic influences on menarcheal timing could not be adjusted for.

## Conclusions

Our data suggest that girls with higher adherence to modern dietary pattern experienced menarche at an earlier age. This association was independent of body mass. Our finding provides evidence to support the recommendation to have a balanced diet for prepubertal girls in China. Further research is needed to address the prospective effects of dietary patterns on earlier stage of pubertal development in Chinese girl population, and to determine the underlying biologic mechanisms.

## Data Availability

The data supporting the findings of this study are available from CHNS (https://www.cpc.unc.edu/projects/china/data/datasets).

## Abbreviations

CHNS: China Health and Nutrition Survey
BMI: Body mass index
FCT: Food Composition Tables
PCA: Principal components analysis
HR: Hazard ratio
CI: Confidence interval
